# Double‐vs single‐balloon catheter for induction of labor: Systematic review and individual participant data meta‐analysis

**DOI:** 10.1111/aogs.14626

**Published:** 2023-07-07

**Authors:** Morgan D. Peel, Doortje M. R. Croll, Jørg Kessler, Birte Haugland, Craig E. Pennell, Jan E. Dickinson, Raed Salim, Noah Zafran, Kirsten R. Palmer, Ben W. Mol, Wentao Li

**Affiliations:** ^1^ Department of Obstetrics and Gynecology Monash University Clayton Victoria Australia; ^2^ Wilhelmina Children's Hospital Birth Center University Medical Center Utrecht Utrecht The Netherlands; ^3^ Department of Obstetrics and Gynecology Haukeland University Hospital Bergen Norway; ^4^ Department of Clinical Science University of Bergen Bergen Norway; ^5^ Voss Hospital Vossevangen Norway; ^6^ School of Medicine and Public Health The University of Newcastle Newcastle Australia; ^7^ Division of Obstetrics and Gynecology The University of Western Australia Perth Australia; ^8^ Department of Obstetrics and Gynecology Emek Medical Center Afula Israel; ^9^ Rappaport Faculty of Medicine, Technion Haifa Israel

**Keywords:** Cook balloon, Foley catheter, individual participant data, induction of labor, safety, systematic review, vaginal birth

## Abstract

**Introduction:**

Evidence comparing double‐balloon vs single‐balloon catheter for induction of labor is divided. We aim to compare the efficacy and safety of double‐vs single‐balloon catheters using individual participant data.

**Material and methods:**

A search of Ovid MEDLINE, Embase, Ovid Emcare, CINAHL Plus, Scopus, and clinicaltrials.gov was conducted for randomized controlled trials published from March 2019 until April 13, 2021. Earlier trials were identified from the Cochrane Review on Mechanical Methods for Induction of Labour. Randomized controlled trials that compared double‐balloon with single‐balloon catheters for induction of labor in singleton gestations were eligible. Participant‐level data were sought from trial investigators and an individual participant data meta‐analysis was performed. The primary outcomes were rates of vaginal birth achieved, a composite measure of adverse maternal outcomes and a composite measure of adverse perinatal outcomes. We used a two‐stage random‐effects model. Data were analyzed from the intention‐to‐treat perspective.

**Results:**

Of the eight eligible randomized controlled trials, three shared individual‐level data with a total of 689 participants, 344 women in the double‐balloon catheter group and 345 women in the single‐balloon catheter group. The difference in the rate of vaginal birth between double‐balloon catheter and single‐balloon catheter was not statistically significant (relative risk [RR] 0.93, 95% confidence interval [CI] 0.86–1.00, *p* = 0.050; *I* ^2^ 0%; moderate‐certainty evidence). Both perinatal outcomes (RR 0.81, 95% CI 0.54–1.21, *p* = 0.691; *I* ^2^ 0%; moderate‐certainty evidence) and maternal composite outcomes (RR 0.65, 95% CI 0.15–2.87, *p* = 0.571; *I* ^2^ 55.46%; low‐certainty evidence) were not significantly different between the two groups.

**Conclusions:**

Single‐balloon catheter is at least comparable to double‐balloon catheter in terms of vaginal birth rate and maternal and perinatal safety outcomes.

## Graphical abstract

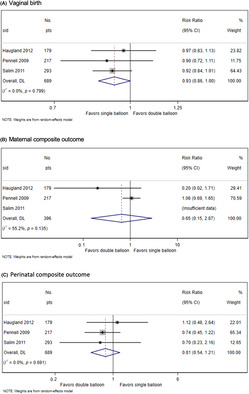

**Abbreviations**
AD‐MA: aggregated‐data meta‐analyses
IPD‐MA: individual participant data meta‐analysis
RCT: randomized controlled trial

**Key message**

Single‐balloon catheter is at least comparable to double‐balloon catheter in terms of vaginal birth rate. Maternal and perinatal safety outcomes are comparable between the two types of balloon catheters.

## 1 INTRODUCTION

Labor induction rates have been increasing globally. In Australia alone, there has been a 20% surge in labor induction rates among selected first‐time mothers from 26% in 2010 to 46% in 2020.^1^ In the presence of an unfavorable cervix for birth, the chances of successful labor induction can be augmented by cervical ripening,^2^ where either mechanical or pharmacological methods are employed.

Mechanical methods mainly include double‐ and single‐balloon catheters. Balloon catheters place pressure on and stretch the cervix, causing a release of local prostaglandins that hasten the cervical ripening process.^2^ A single‐balloon catheter is a Foley catheter inserted into the internal os and the balloon is inflated with 30–80 mL of saline or sterile water^3^ whereas a double‐balloon catheter features two balloons, one inserted just above the cervix and one just below the cervix in the vagina, both balloons are inflated with saline or sterile water.^4^

A multitude of randomized controlled trials (RCTs) have compared double‐balloon with single‐balloon catheters for induction of labor regarding efficacy at achieving vaginal birth and maternal and perinatal safety outcomes, but their findings are divided.^5^, ^6^, ^7^, ^8^, ^9^, ^10^, ^11^, ^12^ In relation to efficacy, two studies recommend double‐balloon catheters,^5^, ^6^ two studies recommend single‐balloon catheters,^7^, ^11^ and four studies recommend that the two methods are comparable.^8^, ^9^, ^10^, ^12^ In relation to safety outcomes no clear patterns were detected.^5^, ^6^, ^7^, ^8^, ^9^, ^10^, ^11^, ^12^ Across aggregated‐data meta‐analyses (AD‐MA) in relation to both efficacy and safety, no significant differences in safety between double‐ and single‐balloon catheters have been detected.^13^, ^14^, ^15^, ^16^ AD‐MA cannot verify the validity of data or properly assess individual safety outcomes for this comparison because events are rare and reporting is inconsistent between trials.

These issues could potentially be solved by performing an individual participant data meta‐analysis (IPD‐MA). IPD‐MA can reanalyze data using the same statistical measure,^17^ assess unreported outcomes,^18^ check data integrity,^18^ allow for in‐depth subgroup analysis,^17^ and generate composite outcomes for safety measures.

We performed this IPD‐MA to compare double‐balloon catheters with single‐balloon catheters for labor induction in relation to efficacy and safety to assist in providing greater clinical clarity.

## 2 MATERIAL AND METHODS

### 2.1 Registration and ethical approval

This IPD‐MA was prospectively registered in PROSPERO (registration number CRD42021226744) and was reported in accordance with the Preferred Reporting Items for Systematic Review and Meta‐Analyses of Individual Participant Data (PRISMA‐IPD) guidelines. Ethical approval was gained from Monash University Human Research Ethics Committee (Project ID 27401).

### 2.2 Search strategy

Eligible trials for inclusion were identified from their inclusion in the Cochrane Review on Mechanical Methods for Induction of Labour^16^ for studies published before March 2019. A further search of the databases Ovid MEDLINE, Embase via Ovid, Ovid Emcare, CINAHL Plus, Scopus, and clinicaltrials.gov was conducted to identify trials published since the Cochrane Review search and was completed on April 13, 2021.

The search strategy keywords included “single balloon”, “foley”, “double balloon”, “atad” and “cook” were combined with MeSH terms and Boolean operators to identify RCTs comparing double‐balloon catheter and single‐balloon catheter for labor induction (Supporting Information Appendix S1).

### 2.3 Study selection

Studies were included if they were an RCT comparing double‐balloon catheter and single‐balloon catheter for labor induction in viable singleton pregnancies. No restrictions were placed on language and non‐English studies were translated online. Two investigators (MP and DC) autonomously reviewed titles and abstracts, and full texts for inclusion following the inclusion and exclusion criteria using the online platform Covidence.^19^ Any conflicts were resolved by a third reviewer (BM).

### 2.4 Data extraction

Corresponding or primary authors were invited to share raw data via email correspondence. If there was no response, co‐authors were invited to participate via email. If also unresponsive, the institutions associated with the authors including hospitals and universities, co‐authors on more recent publications with the author of interest, colleagues from our network in the same country as the author of interest, and finally the journal where the eligible RCT was published were all contacted. A maximum of 12 rounds of emails were sent and corresponding or primary authors were contacted in every round of email.

A data‐sharing agreement between our institution and the corresponding institution was completed before data sharing. Data were transferred via email in password‐protected worksheets and all data shared were deidentified. All data were checked for discrepancies and all discrepancies were clarified with the author of the data set. Data checking also included assessing for missing or excluded data, checking for errors, checking for the presence of randomization, checking internal consistency, and replicating the baseline outcomes and results from the published trial with the trial's raw data.

### 2.5 Outcomes

Primary outcomes include vaginal birth rate, a composite measure of adverse maternal outcomes and a composite measure of adverse perinatal outcomes. Maternal composite outcomes comprised maternal admission to the intensive care unit, maternal infection (defined as a temperature ≥38°C at any time during labor or delivery, antibiotic use or clinically diagnosed infection, such as endometritis), postpartum hemorrhage >1000 mL, maternal death, and uterine rupture. Perinatal composite outcomes include stillbirth (defined as death of a fetus after 20 weeks of gestation or weighing at least 400 g if gestational age unknown), neonatal death (defined as death of a neonate less than 28 days after birth), neonatal seizures, neonatal intensive care unit admission for longer than 48 hours, severe neonatal respiratory compromise (which included mechanical ventilation, infantile respiratory distress syndrome, and pneumothorax), and meconium aspiration syndrome.

Secondary outcomes were selected to further assess the efficacy and safety profiles of the two methods for both the mother and the neonate. They included unassisted vaginal birth rate, assisted vaginal birth rate, emergency and scheduled cesarean section rate, indication for cesarean section and instrumental vaginal birth, time from commencement of cervical ripening to birth, use of oxytocin augmentation, maternal use of analgesia during labor (epidural or spinal), uterine hyperstimulation (defined as either tachysystole or hypertonus with a non‐reassuring fetal heart‐rate pattern on cardiotocography), failed induction (use of another induction method because the original allocated method was not successful), neonatal Apgar score <7 at 5 min, meconium‐stained liquor, and increase in modified Bishop score (difference in modified Bishop score before and after cervical ripening).

### 2.6 Assessment of risk of bias

Two investigators (MP and DC) independently conducted risk‐of‐bias assessments on participating RCTs using the Cochrane Risk of Bias 2 Tool and certainty of evidence assessments using the GRADE approach.^20^ All disagreements were resolved through discussion with a third reviewer (WL) to reach a consensus. The domains assessed for the risk‐of‐bias assessments were randomization process, deviations from intended interventions, missing outcome data, measurement of outcome, and selection of the reported result, and they were assessed for assignment to intervention. Certainty of evidence was assessed for all primary outcomes.

### 2.7 Data synthesis

A two‐stage random‐effects model was used to perform evidence synthesis. All analysis was conducted on an intention‐to‐treat basis. Stage one analysis involved calculating the primary and secondary binary outcomes using relative risks (RR) with 95% confidence intervals (CI) for each trial. To calculate the secondary outcome, change in modified Bishop score, means and standard errors were calculated for each trial. Time‐to‐event analysis for induction to vaginal birth time was calculated using a subdistribution hazard competing risks model. Subdistribution hazard ratios and 95% CI were estimated. Stage two analysis involved combining the summary estimates from stage one analysis using a random‐effects model with the Der Simonian Lair method. Statistical heterogeneity was quantified using *I* ^2^.

Treatment–covariate interaction analysis was performed for the primary outcome vaginal birth rate. Initial subgroups of interest for interaction analysis included gestational age, parity, premature rupture of membranes, and indication for induction. However, there were insufficient data on premature rupture of membranes and indication for induction. We then added body mass index, maternal age, and initial modified Bishop score as explorative covariates. Interaction analysis was performed using generalized linear methods with Poisson regression (robust error estimate) between treatment and covariates for each study. RRs for the interaction terms were then pooled with a random‐effects model, in the same way as the primary analysis. A post hoc subgroup analysis was performed for indication of labor induction and Bishop score (≤3 and ≥4).

To assess for data unavailability bias, AD‐MA was performed for the primary outcome vaginal birth rate for all eligible trials. All analysis was conducted with the Stata version 16.1 (Stata Corp., College Station, TX, USA). All results are presented as double‐balloon catheter compared with single‐balloon catheter (i.e. reference group).

## 3 RESULTS

### 3.1 Study selection

**FIGURE 1:**
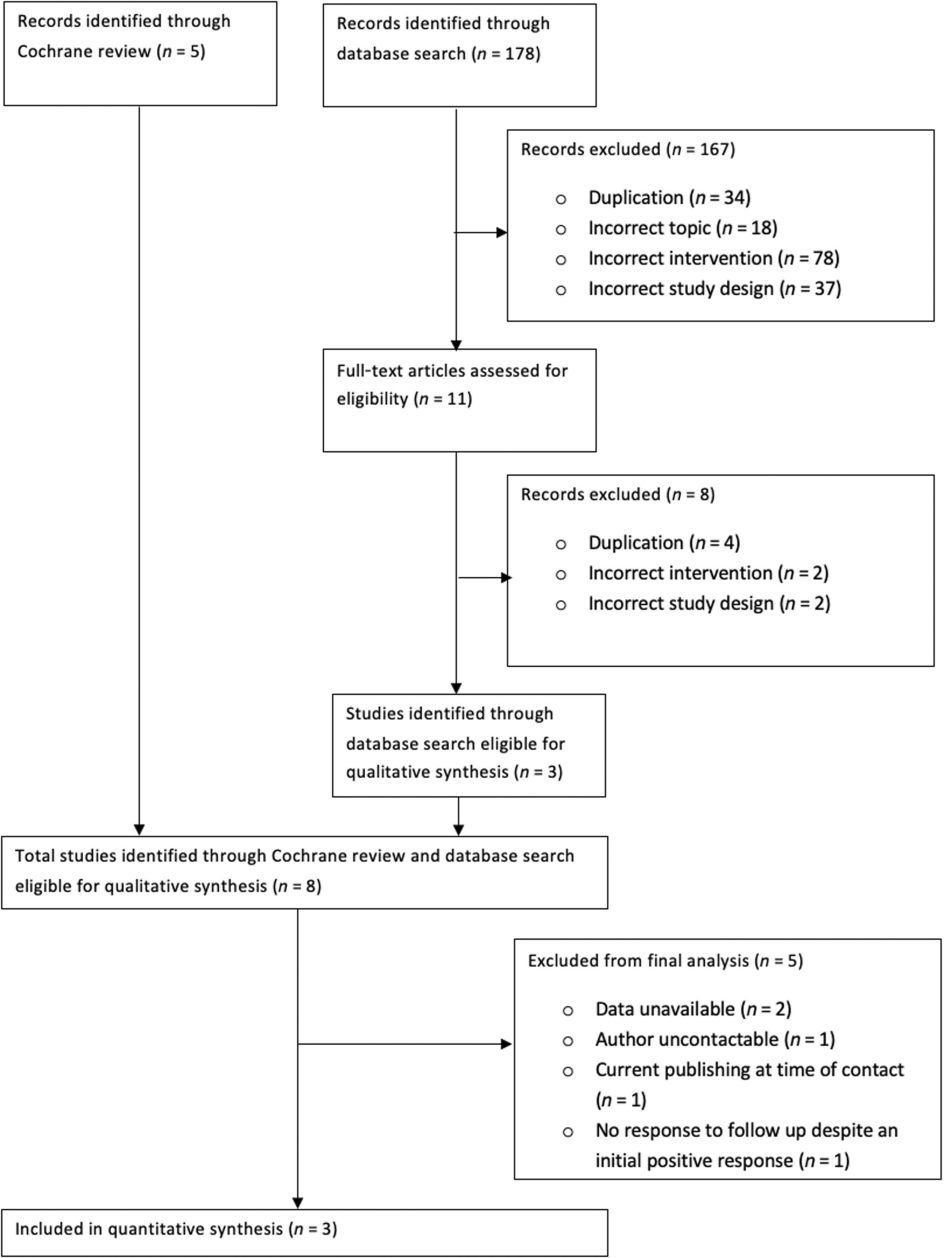
Flowchart summarizing inclusion in individual participant data meta‐analysis of randomized controlled trials comparing double‐balloon catheter with single‐balloon catheter for induction of labor.

Five trials were identified for participation from the Cochrane Review on Mechanical Methods for Induction of Labour^16^ with a further three eligible RCTs, from 178 potentially eligible trials identified since the Cochrane Review^16^ was published (Figure 1). Five eligible studies did not contribute to this IPD‐MA, of which two declared data were unavailable (278 women; albeit published in 2016 and 2019, respectively),^5^, ^6^ one did not respond to all contacts (106 women),^12^ one was seeking publication at the time of contact and gave no response after publication (222 women),^8^ and one did not respond to follow up despite an initial positive response (78 women).^7^ The three participating studies reported on a total of 689 women,^9^, ^10^, ^11^ of which 344 women had been randomized to a double‐balloon catheter and 345 women had been randomized to a single‐balloon catheter.

### 3.2 Study characteristics

A summary of methodological criteria and inclusion/exclusion criteria of eligible studies is available in Supporting Information Appendix S2. The three participating studies were conducted in Norway,^10^ Australia,^11^ and Israel.^9^ The five studies that did not share data were conducted in the USA,^5^ Israel,^6^ Egypt,^7^ Turkey,^8^ and China.^12^ Studies that did not share data (published between 2016 and 2021) were more recent than studies that shared data (published between 2009 and 2011). Non‐shared studies had less clarity on their eligibility criteria.

A summary of the characteristics of participating studies is provided in Table 1. Across participating studies, most participants were nulliparous women with one study recruiting solely nulliparous women.^11^ Data on body mass index were not available in one study.^11^ Median gestational ages and pre‐induction Bishop scores were comparable across participating studies. Mean maternal age was 30.01,^10^ 26.63,^11^ and 28.98^9^ years across contributing trials.

**TABLE 1:** Participants' characteristics of included randomized controlled trials comparing double‐balloon catheter and single‐balloon catheter for induction of labor.

Characteristics	Haugland (2012)	Pennell (2009)	Salim (2011)
Number of participants, *n*	179	217	293
Maternal age (years), mean ± SD	30.01 ± 5.24	26.63 ± 6.05	28.98 ± 5.83
Gestational age (weeks), median (IQR)	40.00 (39.00–42.00)	40.00 (38.00–41.00)	39.00 (38.00–40.00)
BMI, kg/m^2^, mean ± SD	29.03 ± 5.93	Data not collected	24.95 ± 5.13
Parity, *n* (%)			
Nulliparous	117 (65.4%)	217 (100%)	155 (52.9%)
Multiparous	62 (34.6%)	0	138 (47.1%)
Initial modified Bishop score, median (IQR)	3.00 (2.00–4.00)	3.00 (2.00–4.00)	3.00 (2.00–4.00)
Maternal ethnicity, *n* (%)	*n* = 147^a^		
African American	0	Data not collected	Data not collected
Asian	0	Data not collected	Data not collected
Caucasian	141 (78.8%)	Data not collected	Data not collected
Hispanic	0	Data not collected	Data not collected
Other	6 (4.1%)	Data not collected	Data not collected
Indication for labor induction, *n* (%)			
Hypertensive disorders of pregnancy	38 (22.2%)	58 (26.7%)	Data not collected
Post‐term pregnancy	23 (13.5%)	85 (39.2%)	Data not collected
Gestational diabetes mellitus	8 (4.7%)	8 (3.7%)	Data not collected
Oligohydramnios	22 (12.9%)	1 (0.5%)	Data not collected
Suspected intrauterine growth restriction	9 (5.3%)	14 (6.5%)	Data not collected
Other	32 (18.7%)	32 (14.8%)	Data not collected
Two or more indications	39 (22.8%)	19 (8.8%)	Data not collected

Abbreviations: BMI, body mass index; IQR, interquartile range; SD, standard deviation.

^a^ This baseline characteristic was not recorded for all participants.

### 3.3 Risk of bias of included studies

**FIGURE 2:**
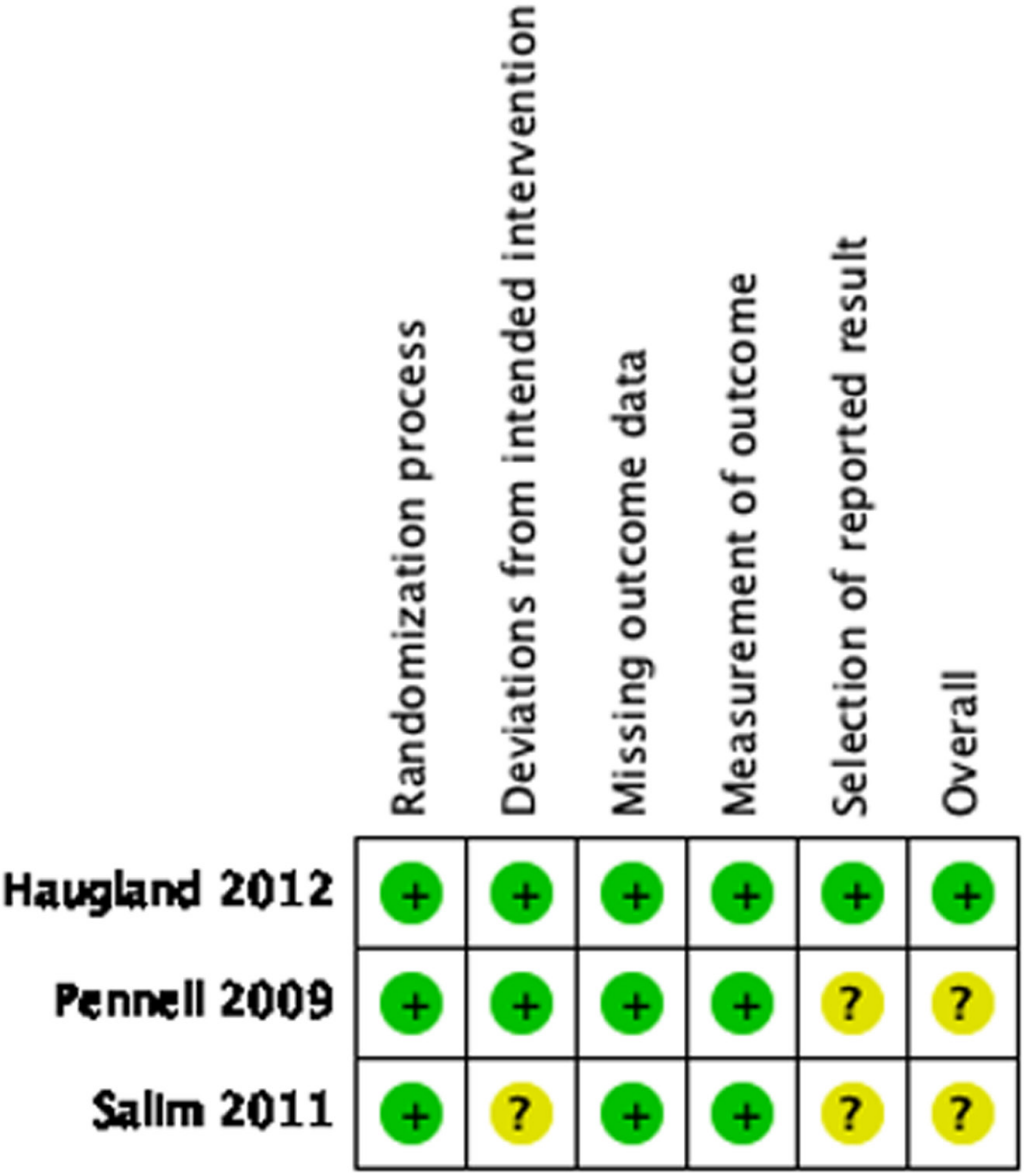
Results of risk of bias assessment of randomized controlled trials comparing double‐balloon catheter to single‐balloon catheter for induction of labor. 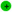, denotes low risk; 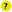; denotes some concerns.

All trials had a low risk of bias for the domain's randomization process, missing outcome data and measurement of outcome. One trial^9^ scored some concerns for the domain deviations from the intended intervention as it was not reported whether an intention‐to‐treat analysis was employed. Two studies^9^, ^11^ for the domain selection of reported results did not include statistical analysis plans and were considered to have some concerns for this domain. (Figure 2).

### 3.4 Synthesis of results

**FIGURE 3:**
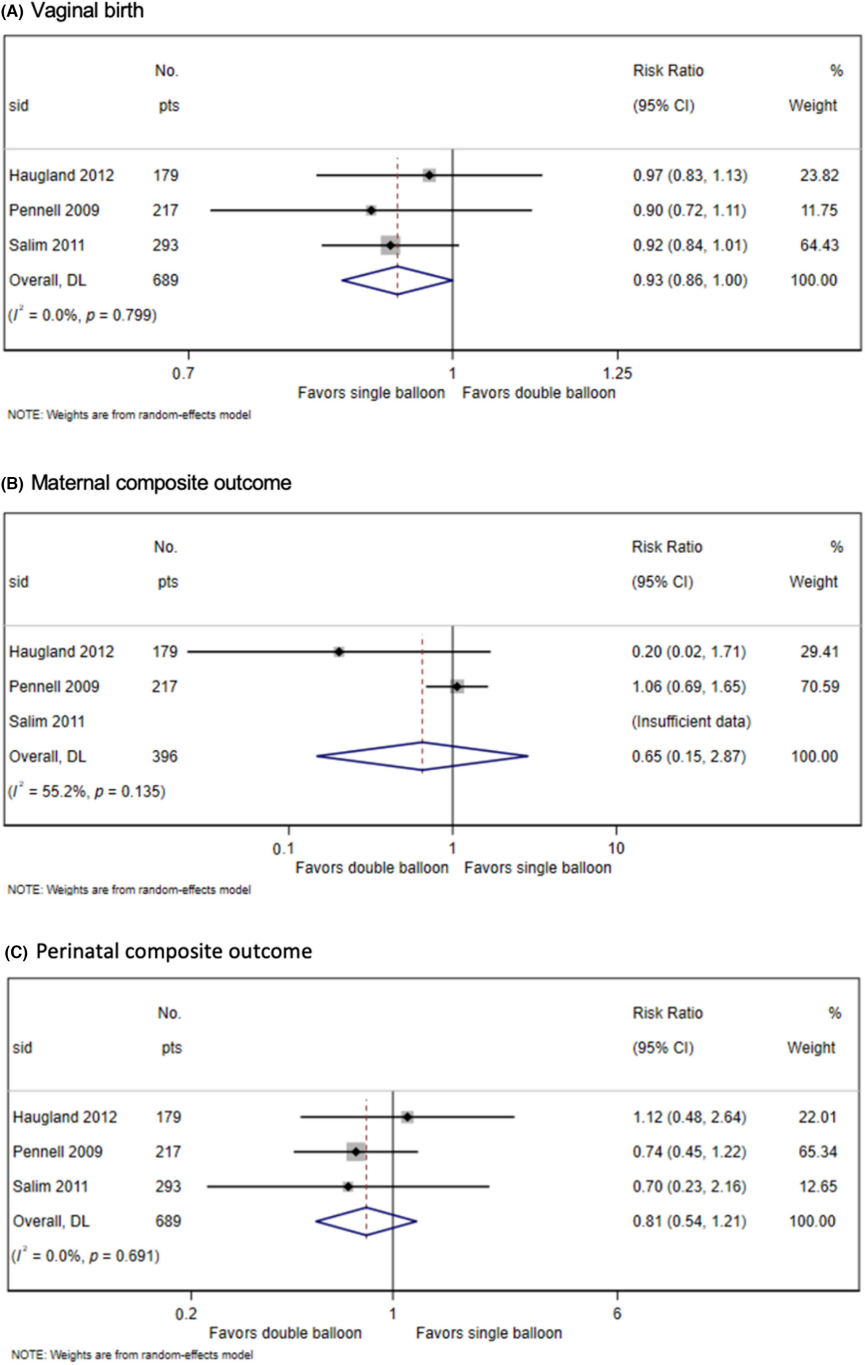
Forest plots showing results of individual participant data meta‐analysis of randomized controlled trials comparing double‐balloon catheter and single‐balloon catheter for induction of labor: (A) vaginal birth, (B) maternal composite outcome, and (C) perinatal composite outcome.

All three studies contributed data to the primary outcomes vaginal birth and maternal composite outcomes, though only two studies contributed data to the perinatal composite outcome. The difference in the rate of vaginal birth between double‐balloon catheter and single‐balloon catheter was not statistically significant, although a trend favorable to single‐balloon catheter was found (crude incidence 73.3% vs 78.8%; RR 0.93, 95% CI 0.86–1.00, *p* = 0.050; *I* ^2^ 0%; moderate‐certainty evidence as downgraded because of imprecision) (Figure 3A). No significant difference between the double‐balloon or single‐balloon catheter in the maternal composite outcome (crude incidence 9.0% vs 9.9%; RR 0.65, 95% CI 0.15–2.87, *p* = 0.571; *I* ^2^ 55.46%; low‐certainty evidence as downgraded because of imprecision and heterogeneity) or perinatal composite outcome (crude incidence 10.5% vs 13.0%; RR 0.81, 95% CI 0.54–1.21, *p* = 0.691; *I* ^2^ 0%; moderate‐certainty evidence as downgraded because of imprecision) was noted (Figure 3B,C).

For secondary outcomes, there was a significantly reduced chance of unassisted vaginal birth associated with double‐balloon catheter (RR 0.87, 95% CI 0.78–0.96, *p* = 0.007; *I* ^2^ 0%). The risk of assisted vaginal birth rate (RR 1.14, 95% CI 0.83–1.58, *p* = 0.416; *I* ^2^ 0%) was similar for both double‐ and single‐balloon catheters (Table 2).

**TABLE 2:** Summary of secondary outcomes results of individual participant data meta‐analysis comparing double‐balloon catheter with single‐balloon catheter for induction of labor.

Outcomes	Double‐balloon catheter (*n*/*N*, No. of trials)	Foley catheter (*n*/*N*, No. of trials)	RR (UCL, LCL)	*p* value	*I* ^2^ statistic (%)
Unassisted vaginal birth rate	190/344, 3 trials	218/345, 3 trials	0.87 (0.78–0.96)	0.007	0
Assisted vaginal birth rate	62/344, 3 trials	54/345, 3 trials	1.14 (0.83–1.58)	0.416	0
Cesarean section for failure to progress	50/344, 3 trials	32/345, 3 trials	1.83 (0.87–3.87)	0.113	45.5
Cesarean section for fetal distress	27/344, 3 trials	25/345, 3 trials	1.09 (0.65–1.82)	0.750	0
Oxytocin use during labor	44/196, 2 trials	36/200, 2 trials	1.20 (0.83–1.74)	0.326	0
Maternal analgesia	163/237, 2 trials	152/235, 2 trials	1.03 (0.92–1.15)	0.631	28.99
Uterine hyperstimulation	0/107, 1 trial	0/110, 1 trial	Insufficient data.		
Apgar <7 at 5 min^a^	2/344, 3 trials	4/34, 3 trials	Insufficient data.		
Meconium‐stained liquor	0 trial	0 trial	Insufficient data.		
	Double‐balloon catheter	Foley catheter	MD (UCL‐LCL)	*p* value	*I* ^2^ statistic (%)
Increase in modified Bishop score	*n* = 221, 2 trials^a^	*n* = 215, 2 trials^a^	0.23 (−0.16–0.62)	0.240	0

Abbreviations: LCL, lower confidence level; MD, mean difference; RR, relative risk; UCL, upper confidence level.

^a^ There were missing data for this outcome.

There was no significant difference in the increase in Bishop score from balloon insertion to removal (mean difference 0.23, 95% CI −0.16 to 0.62; *p* = 0.240; *I* ^2^ 0%). Outcomes including uterine hyperstimulation and rates of meconium‐stained liquor were only reported in one RCT and no positive cases were recorded in either group.^11^ Similarly, maternal infections could not be analyzed because no study recorded data for this outcome.^9^, ^10^, ^11^ There were too few cases of Apgar score <7 at 5 min to power the meta‐analysis for this outcome. The raw incidences were 0.58% and 1.17% in the double‐balloon catheter group and single‐balloon catheter group, respectively.

On pooling subdistribution hazard ratios, the difference in the chance of vaginal birth that considered the time from induction to birth was not significant (subdistribution hazard ratios 0.89, 95% CI 0.73–1.09, *p* = 0.152; *I* ^2^ 0%). A cumulative incidence function that compares the two groups alongside time is shown in Supporting Information Appendix S3.

There were no data for secondary outcomes including indications for instrumental vaginal birth, emergency and scheduled cesarean section, and failed induction.

Covariates including gestational age, parity, body mass index, maternal age, and initial modified Bishop score did not demonstrate statistically significant interactions with treatment for vaginal birth rate. Maternal ethnicity was not analyzed as only one study provided data for this subgroup^10^ (Table 3). In the post hoc subgroup analysis, no significant difference in vaginal birth rate was found in those who received labor induction because of hypertensive disorders of pregnancy (RR 0.95, 95% CI 0.80–1.13, *p* = 0.565; *I* ^2^ = 0%) or post‐term pregnancy (RR 1.00, 95% CI 0.73–1.37, *p* = 0.986; *I* ^2^ = 0%). Other indications did not have sufficient data for the subgroup analysis. Similarly, vaginal birth rate was not significantly different between the two methods in those with Bishop score ≤3 (RR 0.90, 95% CI 0.82–1.00, *p* = 0.055; *I* ^2^ = 0%) or Bishop score ≥4 (RR 0.95, 95% CI 0.85–1.06, *p* = 0.368; *I* ^2^ = 0%).

**TABLE 3:** Summary of intervention–covariate interaction of individual participant data meta‐analysis comparing double‐balloon catheter with single‐balloon catheter for induction of labor.

Characteristics	Number of RCTs	Number of participants	Interaction RR (95% CI)^b^	*I* ^2^ (%)
Gestational age^a^				
<37 weeks	3	689	1.00 (0.74–1.36)	24.8
>40 weeks	3		1.06 (0.91–1.24)	0
Parity	2	472	1.05 (0.90–1.23)	0
Maternal age	3	689	1.00 (0.99–1.01)	0
BMI	2	447	1.00 (0.98–1.02)	0
Initial modified Bishop score	3	688	1.02 (0.97–1.08)	0

Abbreviations: BMI, body mass index; CI, confidence interval; RCT, randomized controlled trial; RR, relative risk.

^a^ Each level compared to 37–40 weeks.

^b^ Statistically significant interaction is indicated when a 95% CI does not contain one.

We also conducted an AD‐MA including all eligible studies for the primary outcome vaginal birth rate and we grouped the results by trials that did and did not share data (Supporting Information Appendix S4). Compared with the results of the IPD‐MA, AD‐MA of trials that did not share data showed that the double‐balloon catheter marginally increases rates of vaginal birth, but the result was not statistically significant (5 trials; 680 participants; RR 1.13, 95% CI 1.00–1.29, *p* = 0.056; *I* ^2^ 45.1%). The rate of vaginal birth was comparable between the two interventions when all trials were pooled together (RR 1.04, 95% CI 0.94–1.15, *p* = 0.453) with significant heterogeneity (*I* ^2^ 59.4%).

## 4 DISCUSSION

In this IPD‐MA, we did not find a statistically significant difference in vaginal birth rate between the double‐balloon catheter and the single‐balloon catheter. Maternal and perinatal safety outcomes were comparable between the two types of balloon catheters.

A strength of our study includes the relatively equal number of participants that each participating trial contributed. This ensures that our study is not strongly influenced by one large study. A further strength is that in conducting an IPD‐MA, rather than an AD‐MA, we were able to assess for potential errors and bias within contributing studies to a higher standard, analyze unpublished but collected data from trials, perform treatment–covariate interaction analysis, combine outcomes measured across trials with different statistical measures, and calculate composite outcomes which are not possible without IPD. Also, we followed a predefined and registered protocol for this IPD‐MA.

Our study does have some limitations. One of which is data unavailability, which in theory could introduce bias. Eight trials were eligible to participate, contact was made with seven of these trials and three shared data with us. However, these three trials contributed over half of the overall number of eligible participants. This disparity between trials eligible to participate and trials that participated was not isolated to this IPD‐MA alone.^21^, ^22^ We previously demonstrated that trials that did not share data in IPD‐MA have more methodological or trustworthiness issues than those that shared data.^23^ Second, not all trials collected data for all the outcomes of interest that we aimed to assess, for example, one trial did not collect data for change in modified Bishop score.^11^

We found double‐balloon catheter may be less effective than single balloon catheter with reduced rates of vaginal birth using IPD from three trials, but this difference was not statistically significant. Although there were differences in clinical characteristics between the included trials such as the volume of saline used for inflating the Foley catheter and the maximum time allowed for balloon placement, the heterogeneity for vaginal birth and most other outcomes appears to be small.

Four previous AD‐MA on this topic found no significant difference between the two groups for vaginal birth rate.^13^, ^14^, ^15^, ^16^ Two trials that were included in AD‐MAs were eligible but unable to participate in our study because of data unavailability even though they were relatively new.^5^, ^6^ One trial that was included in previous AD‐MAs was eligible but did not participate in this IPD‐MA despite an initial positive response from the lead author and further follow‐up emails without response.^7^ In this sense, we are uncertain about the validity of part of the data in previous AD‐MAs, realizing a large proportion of the underlying data could not be verified. There was no statistical significance for the difference in vaginal birth rates, but the AD‐MA of trials that did not share data showed an estimate opposite to that of studies contributing to the IPD‐MA. Unfortunately, analyzing the raw data of trials that did not share data in this IPD‐MA to explain this discrepancy is impossible.

A unique feature of the double‐balloon catheter is that the dilator vector is applied by two balloons inflated on both sides of the cervix when it is held in place, avoiding the need for traction.^24^ Traction refers to a stretching pressure on the lower uterine segment for cervix ripening and is an integral part of the mechanisms of single‐balloon catheter.^25^ This traction may lead to a higher rate of unassisted vaginal birth associated with the single‐balloon catheter found in this study.

Both maternal and perinatal composite outcomes were comparable between double‐ and single‐balloon catheters. AD‐MAs for Apgar scores <7 at 5 min found no significant difference between the two groups.^14^, ^15^, ^16^ Considering both balloon catheters operate by the same stretching mechanism of the cervix to cause effacement and local release of prostaglandins, their extensively similar safety profiles are expected.

Single‐balloon catheter is substantially cheaper than double‐balloon catheter. Also, the cost of an unassisted vaginal birth is lower than the cost of a cesarean section.^26^ Single‐balloon catheter is at least comparable to double‐balloon catheter and offers associated cost reductions to healthcare services. We recently demonstrated in another IPD‐MA that balloon catheters (single and double combined) and vaginal prostaglandins have comparable cesarean delivery rates and maternal safety profiles, but balloon catheters lead to fewer adverse perinatal events.^27^ Therefore, balloon catheters should be used more widely in labor induction. Considering the findings of this study and the associated costs of interventions, single‐balloon catheter is a better option in the induction of labor, especially in low‐resource settings.

## 5 CONCLUSION

Single‐balloon catheter is at least comparable to double‐balloon catheter for both efficacy and safety. In consideration of its lower cost, single‐balloon catheter should be the preferred mechanical method in labor induction.

## AUTHOR CONTRIBUTIONS

BWM and WL designed the meta‐analysis and were responsible for overseeing all aspects of conduct. MDP, KRP, and WL contributed to various stages of the project including aspects of design, eligibility screening, data extraction, risk of bias assessment, IPD checking, and trial analysis. MDP managed the project and collaborative process, WL carried out data synthesis, KRP and BWM provided clinical oversight, and MDP designed and conducted the literature searches. MDP wrote the manuscript supervised by WL with input from all authors. All authors were involved in the decision to submit the manuscript. All contributing trial investigators had opportunities to comment on the initial scope and the draft protocol. Trial investigators also prepared and supplied data and answered questions about their trials.

## CONFLICT OF INTEREST STATEMENT

BWM declared grants from NHMRC, personal fees from ObsEva, personal fees from Merck, personal fees from Guerbet, and grants from Merck, outside the submitted work. KRP has received research grant funds from Glaxo Smith Kline and consultancy fees from Janssen Pharmaceuticals for section 3, unrelated to this work. WL has received research grant funds from the Norman Beischer Medical Research Foundation, unrelated to this work. All other authors have stated explicitly that there are no conflicts of interest in connection with this article.

## Supporting information


Appendix S1‐S4.

## References

[1] Australian Institute of Health Welfare . National Core Maternity Indicators. AIHW; 2022.

[2] Krammer J , O'Brien WF . Mechanical methods of cervical ripening. Clin Obstet Gynecol. 1995;38:280‐286. 7554595 10.1097/00003081-199506000-00010

[3] Levine LD . Cervical ripening: why we do what we do. Sem Perinatol. 2020;44:151216. 10.1016/j.semperi.2019.151216 31813539

[4] Atad J , Bornstein J , Calderon I , Petrikovsky BM , Sorokin Y , Abramovici H . Nonpharmaceutical ripening of the unfavorable cervix and induction of labor by a novel double balloon device. Obstet Gynecol. 1991;77:146‐152. 1984215

[5] Hoppe KK , Schiff MA , Peterson SE , Gravett MG . 30 mL single‐ versus 80 mL double‐balloon catheter for pre‐induction cervical ripening: a randomized controlled trial. J Mat Fetal Neonat Med. 2016;29:1919‐1925. 10.3109/14767058.2015.1067297 26302817

[6] Solt I , Frank Wolf M , Ben‐Haroush S , Kaminskyi S , Ophir E , Bornstein J . Foley catheter versus cervical double balloon for labor induction: a prospective randomized study. J Mat Fetal Neonat Med. 2019;34:1034‐1041. 10.1080/14767058.2019.1623776 31185762

[7] Sayed Ahmed WA , Ibrahim ZM , Ashor OE , Mohamed ML , Ahmed MR , Elshahat AM . Use of the Foley catheter versus a double balloon cervical ripening catheter in pre‐induction cervical ripening in postdate primigravidae. J Obstet Gynaecol Res. 2016;42:1489‐1494. 27436681 10.1111/jog.13086

[8] Obut M , Balsak D , Sarsmaz K , et al. Double Foley catheter for labor induction: an alternative method. Int J Gynecol Obstet. 2021;155:496‐504. 10.1002/ijgo.13807 34197641

[9] Salim R , Zafran N , Nachum Z , Garmi G , Kraiem N , Shalev E . Single‐balloon compared with double‐balloon catheters for induction of labor: a randomized controlled trial. Obstet Gynecol. 2011;118:79‐86. 21691166 10.1097/AOG.0b013e318220e4b7

[10] Haugland B , Albrechtsen S , Lamark E , Rasmussen S , Kessler J . Induction of labor with single‐ versus double‐balloon catheter – a randomized controlled trial. Acta Obstet Gynecol Scand. 2012;91:84‐85.

[11] Pennell CE , Henderson JJ , O'Neill MJ , McCleery S , Doherty DA , Dickinson JE . Induction of labor in nulliparous women with an unfavorable cervix: a randomized controlled trial comparing double and single balloon catheters and PGE2 gel. Obstet Gynecol Surv. 2009;65:78‐80. 10.1111/j.1471-0528.2009.02279.x 19656148

[12] Xing Y , Li N , Ji Q , Hong L , Wang X , Xing B . Double‐balloon catheter compared with single‐balloon catheter for induction of labor with a scarred uterus. Eur J Obstet Gynecol Reprod Biol. 2019;243:139‐143. 31704530 10.1016/j.ejogrb.2019.10.041

[13] De Los Reyes SX , Sheffield JS , Eke AC . Single versus double‐balloon Transcervical catheter for labor induction: a systematic review and meta‐analysis. Am J Perinatol. 2019;36:790‐797. 30380579 10.1055/s-0038-1675206 PMC9214645

[14] Salim R , Schwartz N , Zafran N , Zuarez‐Easton S , Garmi G , Romano S . Comparison of single‐ and double‐balloon catheters for labor induction: a systematic review and meta‐analysis of randomized controlled trials. J Perinatol. 2018;38:217‐225. 29203813 10.1038/s41372-017-0005-7

[15] Liu X , Wang Y , Zhang F , et al. Double‐ versus single‐balloon catheters for labour induction and cervical ripening: a meta‐analysis. BMC Pregnancy Childbirth. 2019;19:358. 31619189 10.1186/s12884-019-2491-4 PMC6796470

[16] de Vaan MDT , ten Eikelder MLG , Jozwiak M , et al. Mechanical methods for induction of labour. Cochrane Database Syst Rev. 2019;10:CD001233. 31623014 10.1002/14651858.CD001233.pub3 PMC6953206

[17] Riley R , Lambert P , Abo‐Zaid G . Meta‐analysis of individual participant data: rationale, conduct, and reporting. BMJ. 2010;340:c221. 20139215 10.1136/bmj.c221

[18] Stewart LA , Parmar MKB . Meta‐analysis of the literature or of individual patient data: is there a difference? Lancet. 1993;341:418‐422. 8094183 10.1016/0140-6736(93)93004-k

[19] Covidence systematic review software . Melbourne, Australia: Veritus Health Innovation.

[20] Schünemann H , Brożek J , Guyatt G , Oxman A . GRADE handbook for grading quality of evidence and strength of recommendations. Updated October 2013. The GRADE Working Group, 2013. Available from guidelinedevelopment.org/handbook.

[21] Nevitt SJ , Marson AG , Davie B , Reynolds S , Williams L , Smith CT . Exploring changes over time and characteristics associated with data retrieval across individual participant data meta‐analyses: systematic review. BMJ. 2017;357:j1390. 28381561 10.1136/bmj.j1390 PMC5733815

[22] Wang H , Chen Y , Lin Y , Abesig J , Wu IXY , Tam W . The methodological quality of individual participant data meta‐analysis on intervention effects: systematic review. BMJ. 2021;373:n736. 33875446 10.1136/bmj.n736 PMC8054226

[23] Bordewijk EM , Wang R , van Wely M , et al. To share or not to share data: how valid are trials evaluating first‐line ovulation induction for polycystic ovary syndrome? Hum Reprod Update. 2020;26:929‐941. 32935841 10.1093/humupd/dmaa031

[24] Atad J , Hallak M , Ben‐David Y , Auslender R , Abramovici H . Ripening and dilatation of the unfavourable cervix for induction of labour by a double balloon device: experience with 250 cases. BJOG. 1997;104:29‐32. 10.1111/j.1471-0528.1997.tb10644.x 8988692

[25] Sherman DJ , Frenkel E , Tovbin J , Arieli S , Caspi E , Bukovsky I . Ripening of the unfavorable cervix with extraamniotic catheter balloon: clinical experience and review. Obstet Gynecol Surv. 1996;51:621‐627. 8888040 10.1097/00006254-199610000-00022

[26] Negrini R , da Silva Ferreira RD , Guimarães DZ . Value‐based care in obstetrics: comparison between vaginal birth and caesarean section. BMC Pregnancy Childbirth. 2021;21:333. 33902486 10.1186/s12884-021-03798-2 PMC8077850

[27] Jones MN , Palmer KR , Pathirana MM , et al. Balloon catheters versus vaginal prostaglandins for labour induction (CPI collaborative): an individual participant data meta‐analysis of randomised controlled trials. Lancet. 2022;400:1681‐1692. 36366885 10.1016/S0140-6736(22)01845-1

