# Supplementary material for: Double‐vs single‐balloon catheter for induction of labor: Systematic review and individual participant data meta‐analysis
Source: Acta Obstet Gynecol Scand. 2023 Jul 7;102(11):1440–9. doi: 10.1111/aogs.14626 (PMC10577628; doi:10.1111/aogs.14626)

# Appendix 1: Search Strategy

**Ovid Medline, Embase via Ovid, Ovid Emcare**

(single balloon OR foley) AND (double balloon OR atad OR cook) AND ((induce OR inducing OR induction)ADJ3(labour OR labor) OR IOL OR cervical ripening)

Limits: 2019-2021

**CINAHL Plus**

(single balloon OR foley) AND (double balloon OR atad OR cook) AND ((induce OR inducing OR induction)N3(labour OR labor) OR IOL OR cervical ripening)

Limits: 2019-2021

**Scopus**

(“single balloon” OR foley) AND (“double balloon” OR atad OR cook) AND ((induce OR inducing OR induction)W/3(labour OR labor) OR IOL OR “cervical ripening”)

Limits: 2019-2021

**Clinicaltrials.gov**

balloon catheter, induction of labour

# Appendix 2: Characteristics of eligible studies

|  | | **Inclusion and Exclusion Criteria** | | | | **Methodology** | | | | |
| --- | --- | --- | --- | --- | --- | --- | --- | --- | --- | --- |
| **Study** | **Country** | **Parity** | **Gestation** | **Bishop score** | **Multifetal gestation** | **Previous caesarean section** | **Single balloon catheter volume** | **Double balloon catheter volume** | **Maximum catheter insertion time** | **Management after balloon removal** |
| **Shared data** | | | | | | | | | | |
| **Haugland 2012** | Norway | Any | ≥ 37 weeks | Cervical dilatation <2cm | Excluded | Included | 60mL | 80mL | 18hrs | Amniotomy and oxytocin infusion was commenced if labor did not begin. Management was the same regardless of the catheter type. |
| **Pennell 2009** | Australia | Nulliparous | ≥ 36 weeks | 4 or less | Excluded | Excluded | 30mL | 80mL | 12hrs | In women with spontaneous onset of labour, amniotomy was performed after cervical dilatation of 3 cm. Oxytocin augmentation was commenced if there was inadequate progress 4 hours after membrane rupture. Management was standardized regardless of the catheter type. |
| **Salim 2011** | Israel | Any | Not reported | 6 or less | Excluded | Excluded | 60mL | 80mL | 12hrs | Artificial rupture of the membranes and oxytocin infusion was commenced if labor did not begin. Labor progress abnormalities were diagnosed and managed according to the recommendations of the ACOG. Management was standardized regardless of the catheter type. |
| **Did not share data** | | | | | | | | | | |
| **Hoppe 2016** | USA | Any | Not reported | 5 or less | Excluded | Included (only one time) | 30mL | 80mL | 12hrs | Bishop score was reassessed after the removal of the catheter. Further cervical ripening with prostaglandins, initiation of oxytocin, or artificial rupture of membranes were performed at the discretion of the managing physician. |
| **Solt 2019** | Israel | Any | ≥ 37 weeks | Not Included | Excluded | Excluded | 40mL | 80mL | 12hrs | Bishop score was reassessed after the removal of the catheter. Oxytocin was administered and amniotomy was performed if the cervical conditions were adequate. |
| **Sayed Ahmed 2016** | Egypt | Nulliparous | Not reported | 4 or less | Excluded | Excluded | 50mL | 80mL | 12hrs | Artificial rupture of the membranes and oxytocin infusion was commenced if labor did not begin spontaneously after removal or spontaneous expulsion of the catheter. |
| **Obut 2021** | Turkey | Any | >24 weeks | 5 or less | Excluded | Excluded | 80mL | 80mL | 12hrs | Oxytocin infusion was started with the standard dose (5 mIU/min) for patients who were not in labor. |
| **Xing 2019** | China | Multiparous | ‘Term’ pregnancy | Not reported | Not reported | Included | 120mL | 80mL | 12hrs | Did not report |

# Appendix 3: Cumulative incidence function for time to vaginal birth after onset of labour induction using double balloon catheter or single balloon catheter


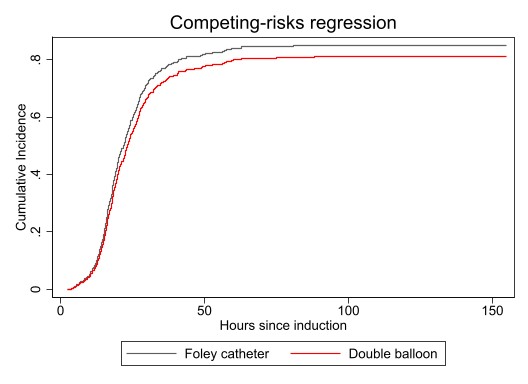


Cumulative incidence function for time to vaginal birth after onset of labour induction using double balloon catheter or single balloon catheter.

# Appendix 4: AD-MA of all eligible trials grouped by whether they shared data or not.


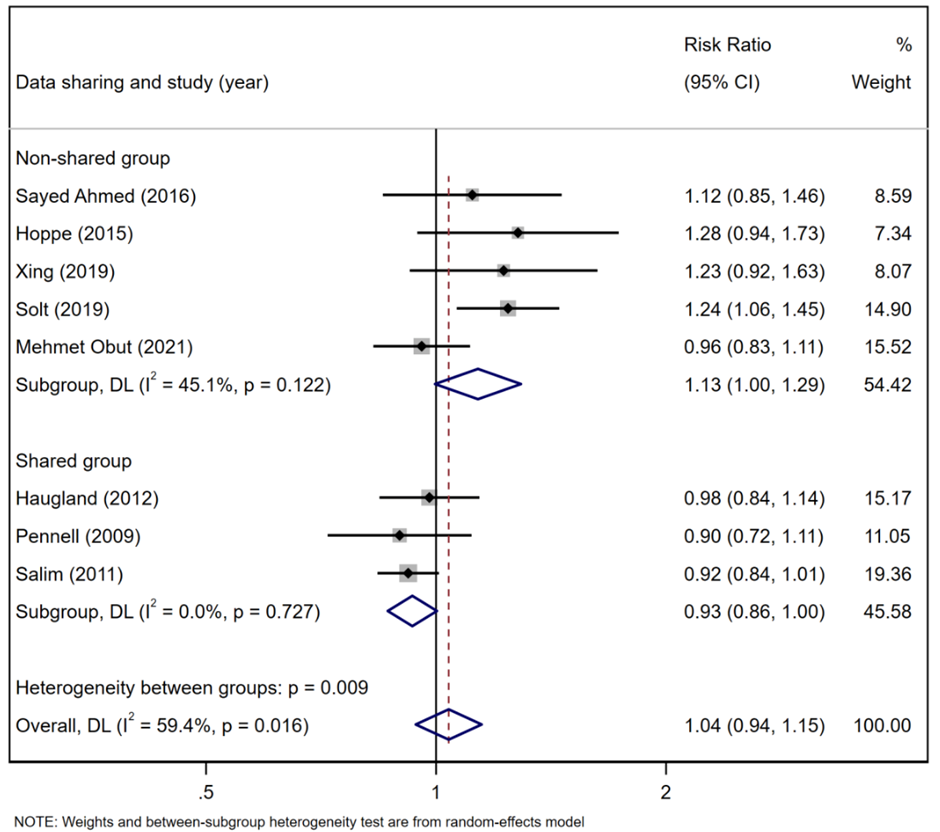

Supplement: Supplementary file 1 — Appendix S1‐S4. [file AOGS-102-1440-s001.docx]
